# Exogenous Glycine Nitrogen Enhances Accumulation of Glycosylated Flavonoids and Antioxidant Activity in Lettuce (*Lactuca sativa* L.)

**DOI:** 10.3389/fpls.2017.02098

**Published:** 2017-12-15

**Authors:** Xiao Yang, Xiaoxian Cui, Li Zhao, Doudou Guo, Lei Feng, Shiwei Wei, Chao Zhao, Danfeng Huang

**Affiliations:** ^1^Key Laboratory of Urban Agriculture (South), Ministry of Agriculture, School of Agriculture and Biology, Shanghai Jiao Tong University, Shanghai, China; ^2^Key Laboratory of Medical Molecular Virology, School of Basic Medical Sciences, Fudan University, Shanghai, China; ^3^Institutes of Biomedical Sciences, Shanghai Medical College, Fudan University, Shanghai, China; ^4^Instrumental Analysis Center, Shanghai Jiao Tong University, Shanghai, China; ^5^Shanghai Agrobiological Gene Center, Shanghai, China; ^6^National Clinical Research Center for Aging and Medicine, Huashan Hospital, Fudan University, Shanghai, China

**Keywords:** luteolin, organic nitrogen, nitrate, quercetin, ascorbic acid, H_2_O_2_ scavenging capability

## Abstract

Glycine, the simplest amino acid in nature and one of the most abundant free amino acids in soil, is regarded as a model nutrient in organic nitrogen studies. To date, many studies have focused on the uptake, metabolism and distribution of organic nitrogen in plants, but few have investigated the nutritional performance of plants supplied with organic nitrogen. Lettuce (*Lactuca sativa* L.), one of the most widely consumed leafy vegetables worldwide, is a significant source of antioxidants and bioactive compounds such as polyphenols, ascorbic acid and tocopherols. In this study, two lettuce cultivars, Shenxuan 1 and Lollo Rossa, were hydroponically cultured in media containing 4.5, 9, or 18 mM glycine or 9 mM nitrate (control) for 4 weeks, and the levels of health-promoting compounds and antioxidant activity of the lettuce leaf extracts were evaluated. Glycine significantly reduced fresh weight compared to control lettuce, while 9 mM glycine significantly increased fresh weight compared to 4.5 or 18 mM glycine. Compared to controls, glycine (18 mM for Shenxuan 1; 9 mM for Lollo Rossa) significantly increased the levels of most antioxidants (including total polyphenols, α-tocopherol) and antioxidant activity, suggesting appropriate glycine supply promotes antioxidant accumulation and activity. Glycine induced most glycosylated quercetin derivatives and luteolin derivatives detected and decreased some phenolic acids compared to nitrate treatment. This study indicates exogenous glycine supplementation could be used strategically to promote the accumulation of health-promoting compounds and antioxidant activity of hydroponically grown lettuce, which could potentially improve human nutrition.

## Introduction

A balanced diet is essential to ensure physical development and health. Numerous epidemiological studies have suggested high daily consumption of fruits and vegetables lowers the risk of several chronic diseases, such as cancer, cardiovascular disease and diabetes; the protective effects of fruit and vegetable consumption are mainly attributed to the presence of bioactive phytochemicals such as polyphenols, vitamin C and vitamin E (Arts and Hollman, 2005; Hooper and Cassidy, 2006; Russo et al., 2012; Chen and Chen, 2013; Wang et al., 2017). The economically valuable vegetable crop lettuce (*Lactuca sativa*) is a minimally processed food product available throughout the entire year, and is a significant source of natural health-promoting compounds. Multiple factors, such as environmental conditions, agronomical manipulation, harvest time, watering and fertilization can strongly influence the levels of health-promoting compounds in horticultural plants (Liu et al., 2007; Li and Kubota, 2009; Becker et al., 2014; Tavarini et al., 2015). Specifically, nitrogen fertilization plays an essential role in balancing the yield and quality of edible plants, especially the levels of secondary metabolites.

As the classical terrestrial nitrogen cycling paradigm asserts that organic nitrogen must be converted into nitrate or ammonium prior to becoming biologically available, the value of organic nitrogen (especially simple forms, such as amino acids) as a fertilizer has been largely ignored (Ge et al., 2009; Näsholm et al., 2009). Recently, several lines of evidence have suggested organic nitrogen (Ge et al., 2009; Gonzalez-Perez et al., 2015), which represents 96–99% of total nitrogen in soil, can be directly absorbed by plants and significantly influence plant physiology and nutritional quality (Paungfoo-Lonhienne et al., 2008). The simple amino acid glycine is regarded as a proof of life, was the original nutrient form for organisms (Xu et al., 2017) and is one of the most abundant free amino acids in horticultural soil; the glycine concentration of soil ranges from 1.14 to 2.39 μg N/g, corresponding to more than 30% of total free amino acids (Wang et al., 2013; Gonzalez-Perez et al., 2015). Compared to other amino acids, there is lower microbial demand for glycine and it is taken up more rapidly by plants (Lipson et al., 1999). Glycine is regarded as a model amino acid in plant organic nitrogen research.

There is growing interest in how nitrogen, especially its inorganic forms, influence antioxidant accumulation and bioactivity. Most studies support the notion that nitrate supply has a negative effect on the biosynthesis of phenolics and vitamin C, as well as antioxidant activity (Lee and Kader, 2000; Awad and de Jager, 2002; Staugaitis et al., 2008; Ibrahim et al., 2012; Yañez-Mansilla et al., 2014). However, recent evidence suggests glycine enhances tolerance to salinity (Badran et al., 2015), drought stress (Yang N. et al., 2016) and cold temperatures (Cao et al., 2017) via elevating the reactive oxygen species (ROS) scavenging system, nitrogen uptake and photosynthesis. In addition, glycine promotes the accumulation of carbohydrates (sucrose, glucose, fructose), which can provide a source of energy and carbon rings for polyphenol biosynthesis (Liu et al., 2016). *L*-phenylalanine, a flavonoid pathway precursor and phenylalanine ammonia lyase (PAL) substrate, was induced in pak choi by exogenous glycine supply (Wang X. L. et al., 2014). We previously assessed the main changes between lettuce cultured in glycine and nitrate without soil using a non-target metabolomics approach. Glycine nitrogen promoted the accumulation of glycosylated quercetin derivatives and luteolin derivatives (quercetin 3-*O*-glucoside, quercetin 3-O-malonylglucoside, luteolin 7-O-glucoside, and luteolin 7-O-glucronide), ascorbic acid and amino acids, but reduced the levels of some phenolic acid derivatives and some organic acids involved in the tricarboxylic acid cycle (Yang et al., 2018). Luteolin 7-O and quercetin 3-O glycosides are potent free radical scavengers/antioxidants and prevent ROS generation effectively (Agati et al., 2012; Brunetti et al., 2013). Therefore, we hypothesized glycine supply could promote the synthesis of health-promoting compounds in lettuce.

Thus, in the present study, the influence of different concentrations of organic nitrogen (as glycine) on the nutritional quality (i.e., total polyphenol, flavone, vitamin C and vitamin E contents, and antioxidative activity) of two lettuce cultivars was determined using a metabolomics approach and *in vitro* bioactivity assays. This work further explores the biological effects of organic nitrogen supply and indicates exogenous supply of glycine could potentially be used to enhance the nutritional quality of lettuce.

## Materials and methods

### Plants and cultivation

**Figure 1:**
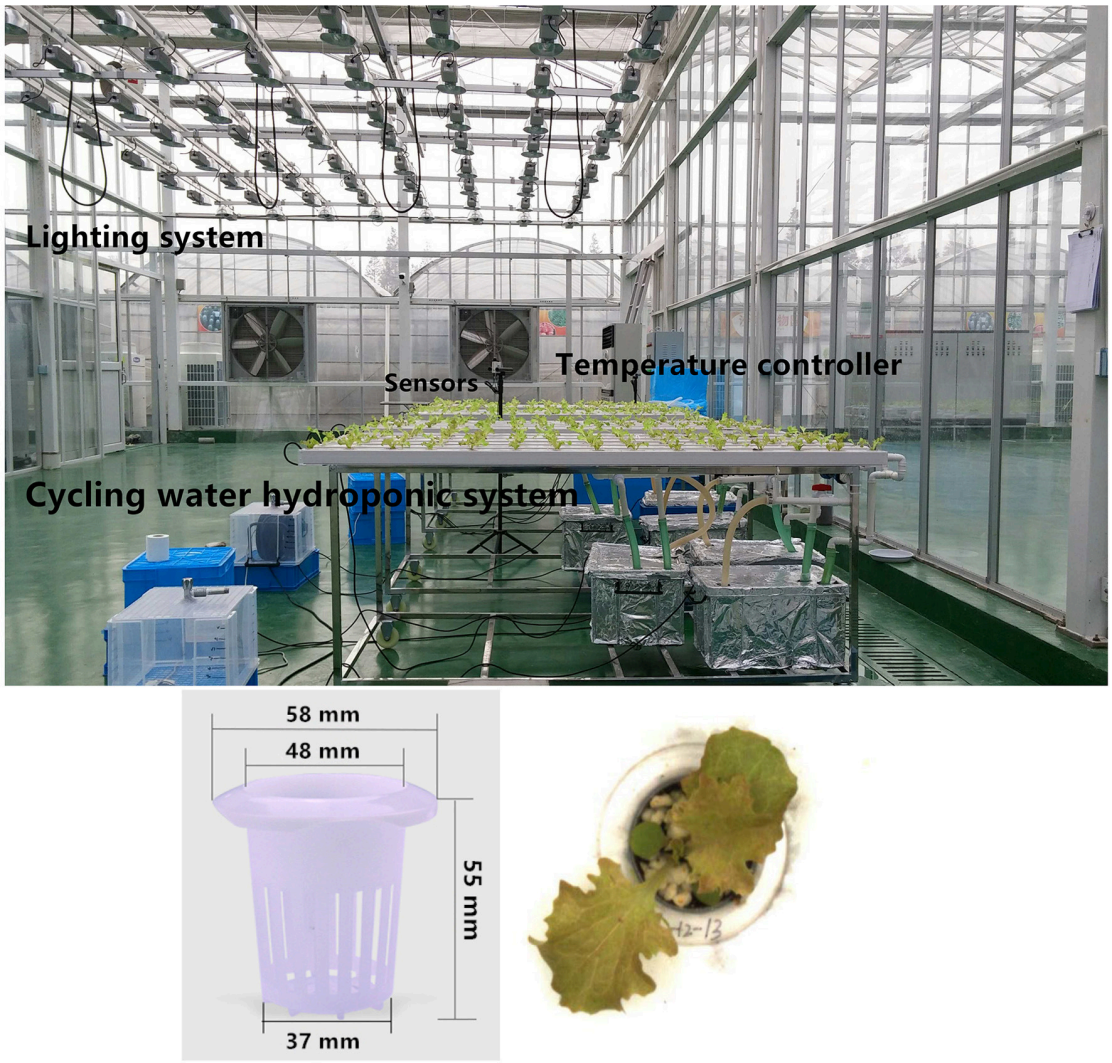
The greenhouse hydroponic apparatus and mesh pot net baskets used to cultivate lettuce.

Seeds of the lettuce cv. Lollo Rossa and cv. Shenxuan 1 were purchased from Atlas Seeds BJ Co., Ltd, (Beijing, China) and the Horticultural Research Institute, Shanghai Academy of Agriculture Sciences (China), respectively. Seeds were sown in white mesh pot net baskets (Figure 1) in nursing substrate (100% perlite) and germinated in a greenhouse at Shanghai Sunqiao Modern Agricultural Development Zone, China (latitude 31°17′ N, longitude 121°62′ E; altitude, 4 m above mean sea level). Then, 21-day-old seedlings were transferred to a water-cycled hydroponic experiment device (Figure 1). After recovering the seedlings in water for 3 days, the seedlings were cultivated for 30 days in nitrogenous nutrient solution (1.25 mM Mg, 3.5 mM K, 1.25 mM S, 2.05 mM Ca, 1 mM P, and 6.6 mM Cl, pH 5.8) containing different forms and concentrations of nitrogen: 9 mM nitrate (as NaNO_3_, control, 9Nit), 4.5 mM glycine (4.5Gly), 9 mM glycine (9Gly) or 18 mM glycine (18Gly). Ampicillin (10 mg/L) was added to the nutrient solution to prevent bacterial infections (Okamoto and Okada, 2004).

All treatments (90 seedlings per treatment, three replicates) were arranged randomly. During the experiment, environmental conditions were maintained at 22 ± 3°C during the day and 15 ± 2°C at night with 250–280 μmol·m^−2^·s^−1^ during the 14 h photoperiod (natural and artificial lighting). The culture solutions were contained in a circulating water system and renewed every 2 days. At the end of cultivation, all leaves were collected. Each sample was divided in two: one half was used for physiological assessments; the other half was flash frozen in liquid nitrogen and stored at −80°C until further analysis.

### Chemicals and reagents

Ultra-pure water was prepared using a Milli-Q system (Millipore Laboratory, Bedford, MA, USA). Methanol and acetonitrile (LC-MS grade) were purchased from Fisher Scientific (Pittsburgh, PA, USA); luteolin 7-glucoside, quercetin glucoside and chicoric acid standards (HPLC grade, ≥98%), from the Chinese National Institute for Food and Drug Control (Beijing, China); methoxyamine hydrochloride, *L*-2-chlorophenylalanine, bis (trimethylsilyl) trifluoroacetamide (BSTFA), and 2′,7′-dichlorofluorescin diacetate (DCFH-DA), from Sigma-Aldrich (Merck KGaA, Darmstadt, Germany); Dulbecco's modified Eagle medium (DMEM) and fetal bovine serum, from Invitrogen (Thermo Fisher Scientific, Waltham, MA, USA); *L*-glutamine and penicillin, from Sangon Biotech (Shanghai, China); the Cell Counting Kit-8, from Dojindo (Kumamoto, Japan); and 2, 2′-azobis (2-amidinopropane) dihydrochloride (ABAP), form Wako (Osaka, Japan). All other chemicals were of analytical grade and obtained from China National Medicines Co., Ltd. (Shanghai, China).

### Analysis of the fresh weight and total phenolic and anthocyanin contents of lettuce leaves

Fresh weight was measured using electronic scales (AUY220; Shimadzu, Kyoto, Japan). For total phenolic content analysis, 1 g of raw leaf was ground in 10 mL of 0.1 mM HCl-methanol (*v/v* 1:1), extracted ultrasonically for 30 min and centrifuged at 9,000 g for 30 min. The supernatant was diluted to 25 mL with methanol and filtered through a 0.45 μm membrane (Złotek et al., 2014). Total polyphenol content was quantified using a UV-Vis spectrophotometer (U2900; Hitachi, Tokyo, Japan) at 725 nm as described by Złotek et al. (2014) and expressed as mg gallic acid equivalent (GAE) per g fresh weight (mg GAE·g ^−1^ FW). The formula for calculating GEA was *Y* (GAE, mg) = 0.1266 × (OD_725nm_) − 0.0008.

Total anthocyanin content was measured via a non-destructive method, as described previously (Yang X. et al., 2016; Ferrandino et al., 2017), using a Dualex 4 Scientific^+^ (Dx4, FORCE-A, Orsay, France) and expressed as: anthocyanin content (ng per cm^2^) = log (red-excited infrared fluorescence/green-excited infrared fluorescence) × 10^3^.

### UPLC-VION-IMS-QTOF-MS/MS analysis

For UPLC-MS, lettuce leaf samples (200 mg) were weighed, ground into a powder in liquid nitrogen, extracted in 1 mL methanol/water (80:20, *v/v*), sonicated at 25°C for 30 min, incubated at 4°C for 12 h, centrifuged at 12,000 g for 10 min, and 0.5 mL supernatant was used for UPLC-MS analysis as previously described (Abu-Reidah et al., 2013a; Yang et al., 2018).

The composition and relative contents of polyphenols in lettuce leaves were analyzed using an Acquity class UPLC and Vion IMS QTOF MS (Waters, Corp. Milford, MA, USA) using an Acquity UPLC HSS T3 column (100 mm × 2.1 mm, i,d.: 1.7 μm). The mobile phases were water containing 0.1% formic acid (A) and acetonitrile containing 0.1% formic acid (B). The injection volume was 3 μL, flow rate was 0.4 mL/min, with gradient elution (0–4 min, 20% B; 4–6 min, linear gradient from 20 to 25% B; 6–8.5 min, 50% B; 8.5–12.5 min 50–85% B, 12.5–14 min, 85–100% B; 14–17 min, 100%), then initial conditions were restored for 5 min to equilibrate the column. The scan range was 50–1,000 *m/z*, and spectra were acquired in negative-ion mode. MS and MS/MS spectra were identified based on accurate mass, MS^2^ fragments and isotopic distribution using online databases (e.g., ResPect, http://spectra.psc.riken.jp/) and bibliographies related to lettuce metabolites. MS and MS/MS tolerance were set at 3 mDa and 10 mDa, respectively. MS and MS/MS data were processed using Progenesis QI software (Waters Corp.).

To evaluate analytical reliability and reproducibly, quality control (QC) samples (a mixture of all samples) were analyzed at the start, middle and end of each batch, as previously described (Want et al., 2013). Principal component analysis (PCA) showed the PCA scores of the seven QC samples clustered together (Supplemental Figure 1), confirming the reliability and repeatability of the metabolomic analysis.

### GC-MS analysis

For GC-MS analysis, 200 mg lettuce leaf tissue was ground in liquid N, extracted in 1 mL ice-cold methanol:chloroform (3:1 *v/v*), 20 μL of 0.3 mg·mL^−1^ *L*-2-chlorophenylalanine (internal standard) was added, the samples were centrifuged at 15,000 g for 10 min, 0.3 mL of the supernatant was vacuum freeze-dried at 25°C, 80 μL methoxyamine hydrochloride (15 mg mL^−1^ in pyridine) was added, incubated at 37°C for 1.5 h, then 80 μL bis (trimethylsilyl) trifluoroacetamide (BSTFA, containing 1% trimethylchlorosilane) was added and incubated at 80°C for 1 h (Du et al., 2011).

Relative GC/MS quantification of ascorbic acid, α-tocopherol and γ-tocopherol were performed using an Agilent 7890 Gas Chromatograph coupled to a LECO Mass Spectrometer (PerkinElmer Inc., Waltham, MA, USA) using a DB-5MS capillary column (30 m × 0.25 mm × 0.25 μm; Agilent J&W Scientific, Folsom, CA, USA). Inlet temperature, transfer line temperature and ion source temperature were 280°C, 280°C, and 230°C, respectively. The gas (helium) flow rate was 1 mL·min^−1^ and injection volume was 1 μL. After 6.5 min solvent delay, the initial GC oven temperature was 60°C; 1 min after injection, the GC oven temperature was raised to 300°C at 5°C/min, then held at 300°C for 11 min. Measurements were made via electron impact ionization (70 eV) in full scan mode (*m/z* 33–600). Ascorbic acid, α-tocopherol and γ-tocopherol were identified using LECO Chroma TOF (PerkinElmer Inc.) by comparison with reference spectra in the NIST 14 Mass Spectral Library (Scientific Instrument Services, Inc. NJ, USA). Relative response ratio was obtained by dividing the peak area by the peak area for *L*-2-chlorophenylalanine.

### Antioxidant bioactivity analysis

#### Reducing potential assay

The antioxidant activity of the lettuce leaf extracts was determined using the ferric-reducing antioxidant power assay (FRAP) (Chan et al., 2007). Samples (2.5 mL, extracted as described for polyphenol analysis) were mixed with 2.5 mL phosphate buffer (0.2 mM, pH 6.6) and 2.5 mL potassium ferricyanide (1% *w/v*), incubated at 50°C for 20 min, and then immediately transferred onto ice. Trichloroacetic acid solution (2.5 mL of 10% *w/v*) was added to stop the reaction, the mixture was centrifuged at 3,000 g for 10 min, then 2.5 mL of the supernatant was diluted with 2.5 mL water, 0.5 mL ferric chloride solution (0.1% *w/v*) was added, incubated for 30 min and absorbance was determined at 700 nm. The extraction solution used for polyphenol analysis was used as control sample. FRAP values were expressed as mg GAE·g ^−1^ FW.

#### Preparation of extracts for cellular antioxidant activity assays

Lettuce leaf extracts were prepared as described for UPLC-MS analysis. Prior to the antioxidant bioactivity assays, 0.5 mL of supernatant from each sample was vacuum freeze-dried at 25°C and resuspended in 200 μL water containing 0.1% DMSO.

#### Cytotoxicity assay

Hepatitis B virus-producing HepG2 cells were cultured in DMEM supplemented with 2 mM *L*-glutamine, 50 U mL^−1^ penicillin and 10% fetal bovine serum at 37°C in a 5% CO_2_ atmosphere (Hong et al., 2013). The cytotoxicity of the lettuce leaf extracts toward HepG2 cells was assessed using the CCK-8 assay, as described by Shi et al. (2017). Cell viability (%) was calculated as (OD450 _(sample)_ – OD450 _(blank)_)/(OD _(mock)_ – OD450 _(blank)_).

#### Cellular antioxidant activity (CAA) assay

Cellular antioxidant activity (CAA) was quantified as previously described (Wolfe and Liu, 2007). Briefly, HepG2 cells were seeded at a density of 1 × 10^5^ cells per well into 96-well microplates in 100 μL media. After 24 h, the media was removed, cells were washed with PBS, then incubated with 100 μL of media containing 25 μM DCFH-DA and 0.5 μL lettuce leaf extract for 1 h at 37°C. Then the solution was removed, 100 μL of 600 μM ABAP was added, and fluorescence values were read at 485 nm excitation and 538 nm emission using a Victor™ X3 Multilabel Plate Reader (Perkin Elmer) every 5 min for 1 h. CAA was expressed as CAA (unit) = 100 – (∫SA /∫CA) × 100, where SA is the integrated area of sample fluorescence vs. time curve and CA is the integrated area of the control curve.

#### Cellular H_2_O_2_ scavenging assay

HepG2 cells were seeded at 5,000 cells/well in 96-well plates in 100 μL media, cultured for 24 h, incubated with 400 μM H_2_O_2_ containing 1 μL of lettuce leaf extract or an equivalent volume of media (mock) or H_2_O_2_ (400 μM H_2_O_2_ solution plus 1 μL medium) as control treatments for 24 h, and cell viability was assessed using the CCK-8 assay as described by Shi et al. (2017).

### Statistical analysis

Values are the mean ± SD of three biological replicates per treatment and three technical replicates per sample. ANOVA based on LSD analysis and Students *t*-tests were performed using IBM SPSS Statistics 22 (IBM, Armonk, NY, USA); *p* < 0.05 was considered significant. Pathway analysis was performed using ProcessOn (https://www.processon.com/) and R software (https://www.r-project.org/). PCA analysis was performed using SIMCA-P13.0 (Sartorius Stedim Biotech, Gottingen, Germany), Pearson correlation analysis was conducted using R software. Figures were created using R software or OriginPro 2016 (OriginLab, Northampton, MA, USA).

## Results

### Effect of glycine on growth of lettuce

The fresh weights of the aboveground parts and whole lettuce plants after 30 days cultivation in hydroponic solution containing 9 mM nitrate (control) or 4.5, 9, or 18 mM glycine are shown in Table 1. Glycine significantly reduced the fresh and aboveground weights compared to control lettuce. Among the glycine-treated plants, 9 mM glycine led to a significantly higher fresh weight (*p* < 0.05) than 4.5 or 18 mM glycine.

**Table 1:** Plant fresh and dry weights and total anthocyanin contents of lettuce cultivated in hydroponic solution containing nitrate or glycine.

**Treatment**	**Shenxuan 1**			**Lollo Rossa**		
	**Fresh weight (g) per plant**	**Above ground fresh weight (g) per plant**	**Total anthocyanidin content (ng per cm^2^)**	**Fresh weight (g) per plant**	**Above ground fresh weight (g) per plant**	**Total anthocyanidin content (ng per cm^2^)**
9Nit	55.90 ± 4.72a	47.70 ± 5.31a	n.d.	43.47 ± 3.72a	35.35 ± 2.88a	6.88 ± 0.81c
4.5Gly	17.98 ± 2.51c	13.85 ± 2.48c	n.d.	16.55 ± 1.51c	12.95 ± 1.82c	10.33 ± 1.21b
9Gly	23.46 ± 1.48b	19.90 ± 1.51b	n.d.	20.88 ± 2.29b	18.26 ± 2.33b	10.61 ± 0.99b
18Gly	14.85 ± 1.05c	11.43 ± 1.94c	n.d.	12.96 ± 1.08c	11.35 ± 1.02c	34.72 ± 2.01a

*For fresh weight analysis, values are means ± SD (n = 4); for total anthocyanidin content, values are mean ± SD (n = 100); Value with different letters are significantly different; p < 0.05, LSD analysis*.

*n.d. represents not detected*.

### Effect of glycine on accumulation of antioxidative compounds

**Figure 2:**
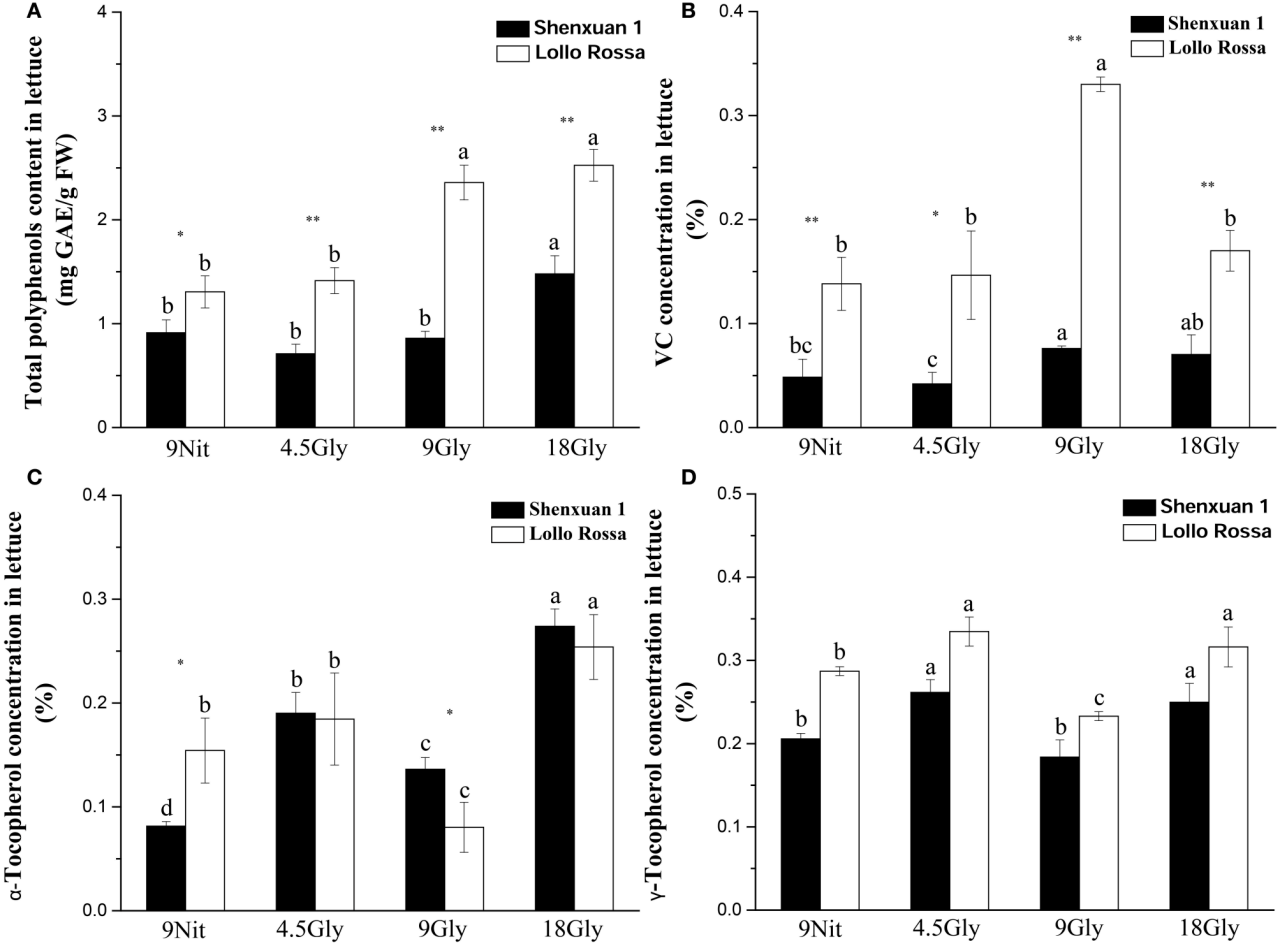
Effect of glycine and nitrate supply on the contents of bioactive compounds in lettuce leaf extracts. **(A)** Total polyphenol content; **(B)** ascorbic acid content; **(C)** α-tocopherol content; **(D)** γ-tocopherol content. Different small letters indicate significant differences, *p* < 0.05; LSD analysis (*n* = 3). The “^*^” (*p* < 0.05) and “^**^” (*p* < 0.01) indicate significance differences between cultivars (*t*-test).

In this study, the total anthocyanidin content was only assessed in the Lollo Rossa cultivar; the Shenxuan 1 cultivar is a green leafy lettuce, which does not contain detectable levels of anthocyanidins. As shown in Table 1, glycine supply increased (*p* < 0.05) the total anthocyanidin content in Lollo Rossa leaves compared to control plants. The anthocyanidin level peaked at 34.72 ng per cm^2^ in Lollo Rossa leaves exposed to 18 mM glycine, which was significantly higher than the plants treated by 4.5 or 9 mM glycine. The highest exogenous concentration of glycine (18 mM) also significantly (*p* < 0.05) increased the total polyphenol content of the lettuce leaves compared to lettuce cultivated in 9 mM nitrate or 4.5 or 9 mM glycine (Figure 2A), with maximal levels of 1.48 and 2.53 mg g^−1^ observed in Shenxuan 1 and Lollo Rossa, respectively.

In addition, relative ascorbic acid content increased significantly as glycine supply increased (from 4.5 to 9 mM glycine), but decreased at 18 mM glycine (Figure 2B) compared to control lettuce. The α-tocopherol content peaked in lettuce exposed to 18 mM glycine, corresponding to respective 3.4- and 1.7-fold increases in Shenxuan 1 and Lollo Rossa compared to the controls supplied with 9 mM nitrate (Figure 2C). Moreover, in both varieties, the levels of γ-tocopherol were significantly higher in lettuce supplied with 4.5 and 18 mM glycine (*p* < 0.05) than control plants (Figure 2D).

**Figure 3:**
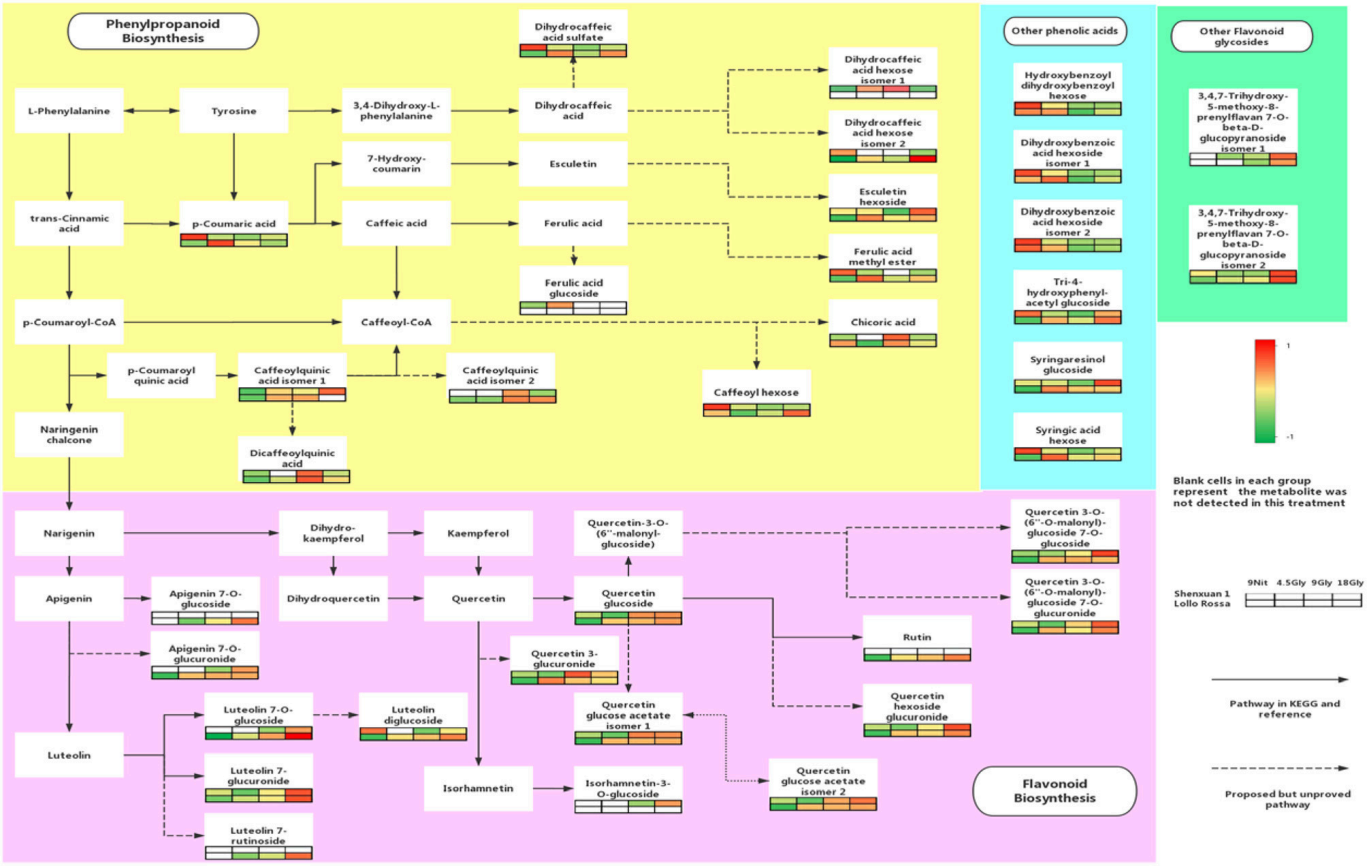
Effect of glycine and nitrate supply on the composition and concentrations of polyphenols in lettuce leaf extracts. Relative abundance of metabolites is indicated from red (high) to green (low). The dotted lines in the metabolic pathway represent possible relationships that have not yet been proven experimentally and solid lines indicate pathways in the KEGG or PlantCyc databases.

UPLC-MS analysis can separate co-effluents and enables robust and reproducible identification of the isomeric structures of polar metabolites (e.g., phenolic compounds) (Paglia et al., 2014). By comparison with online and in-house databases as well as published data, a total of 35 polyphenols were tentatively identified in the lettuce leaf extracts (level 2, putatively-annotated compounds), including 17 phenolic acid derivatives and 18 glycosylated flavonoids (Table 2). Metabolic pathway analysis was conducted to investigate the relationships between glycine supply and the accumulation of phenolic acids and flavonoids (Figure 3).

**Table 2:** Metabolites putatively identified by UHPLC-IMS-QTOF-MS in the leaf extracts of nitrate- and glycine-treated lettuce.

**No**.	**Component name**	**Neutral mass (*m/z*)**	**Observed (*m/z*)^−^**	**Mass Error (mDa)**	**RT (min)**	**Major fragments *m/z* (%)**	**References**
1	Dihydrocaffeic acid hexose isomer 1	344.11073	343.1033	−0.2	1.82	181.05045(100);163.04019(52);135.04524(28)	Abu-Reidah et al., 2013b
2	Dihydroxybenzoic acid hexoside isomer 1	316.07943	315.0723	0.2	1.87	152.01166(33);153.01921(23); 108.02183(21);109.02906(11)	Ammar et al., 2015
3	Dihydrocaffeic acid sulfate	262.01472	261.0073	−0.2	1.96	181.05084(100);163.04070(87);135.04620(75)	Abu-Reidah et al., 2013a
4	Dihydroxybenzoic acid hexoside isomer 2	316.07943	315.0721	0.0	2.02	153.01956(50);109.02965(18)	Ammar et al., 2015
5	Syringic acid hexose	360.10565	359.0984	0.0	2.13	182.02196(100);197.04479(49)	Abu-Reidah et al., 2013b
6	Esculetin hexoside	340.07943	339.0717	−0.5	2.33	177.01914(100);133.02890(10)	Ammar et al., 2015
7	Quercetin-hexoside-glucuronide	640.12756	639.1207	0.4	2.45	463.08834(100);301.03547(20)	Abu-Reidah et al., 2013a
8	Dihydrocaffeic acid hexose isomer 2	344.11073	343.1032	−0.2	2.49	181.05038(100);137.06112(81)	Abu-Reidah et al., 2013a
9	Caffeoyl hexose	342.09508	341.0878	0.0	2.52	135.04502(100);179.03470(36)	Amessis Ouchemoukh et al., 2014
10	Caffeoylquinic acid isomer 1^a^	354.09508	353.0879	0.1	2.52	191.05594(100);135.04502(13)	Clifford et al., 2005; Jaiswal et al., 2011
11	Quercetin 3-O-(6″-O-malonyl)-glucoside 7-O-glucuronide	726.12796	725.1187	−2.0	2.60	505.09931(100);681.13135(26);301.03416(21)	Llorach et al., 2008
12	Quercetin 3-O-(6″-O-malonyl)-glucoside 7-O-glucoside	712.14869	711.1407	−0.7	2.67	505.09860(49);462.08120(23);301.03539(23)	Llorach et al., 2008
13	Ferulic acid methyl ester	208.07356	207.0651	−1.2	2.69	192.04259(100);177.01972(65)	Abu-Reidah et al., 2013a
14	Ferulic acid glucoside	356.11073	355.1029	−0.6	2.77	193.05083(100)	Abu-Reidah et al., 2013c
15	Caffeoylquinic acid isomer 2^a^	354.09508	353.088	0.2	2.83	191.05575(100)	Clifford et al., 2005; Jaiswal et al., 2011
16	p-coumaroylquinic acid	338.10017	337.0931	0.2	3.20	191.05545(100)	Clifford et al., 2006; Abu-Reidah et al., 2013a
17	Luteolin diglucoside	610.15338	609.1459	−0.2	3.17	285.04098(100)	Marques et al., 2009
18	Rutin	610.15338	609.1459	−0.2	3.17	301.03423(35)	Abu-Reidah et al., 2013b
19	Chicoric acid	474.07983	473.0725	−0.1	3.31	135.04460(91);149.00862(48); 179.03440(100);293.02990(88)	Standard
20	Luteolin 7-rutinoside	594.15847	593.1511	0.0	3.48	285.04018(100)	Llorach et al., 2008
21	Quercetin 3-glucuronide	478.07474	477.0678	0.3	3.52	301.03501(100)	Llorach et al., 2008; Rodríguez-Medina et al., 2009
22	Quercetin glucoside	464.09548	463.0883	0.1	3.56	300.02702(100);271.02465(80);255.02969(52)	Standard
23	Luteolin 7-glucuronide	462.07983	461.0721	−0.5	3.59	285.04025(100)	Abu-Reidah et al., 2013b
24	Luteolin 7-O-glucoside	448.10056	447.0927	−0.6	3.63	284.03218(76)	Standard
25	3,4,7-Trihydroxy-5-methoxy-8-prenylflavan 7-O-beta-D-glucopyranoside isomer 1	534.21011	533.2031	0.3	3.64	473.18194(100);491.19196(60)	Abu-Reidah et al., 2013a
26	Isorhamnetin-3-O-glucoside	478.11113	477.1039	0.1	3.76	243.02975(100);299.01988(80)	Schieber et al., 2002
27	Apigenin 7-O-glucuronide	446.08491	445.0773	−0.3	3.76	271.02448(100)	Abu-Reidah et al., 2013b
28	Quercetin glucose acetate isomer 1	506.10604	505.0988	0.1	3.77	300.02729(100);255.02978(29)	ResPect MS/MS
29	Dicaffeoylquinic acid^b^	516.12678	515.1201	0.6	3.93	191.05593(100);353.08798(50)	Clifford et al., 2005
30	Quercetin glucose acetate isomer 2	506.10604	505.0993	0.5	3.94	300.02747(100)	ResPect MS/MS
31	Apigenin 7-O-glucoside	432.10565	431.0994	1.0	4.24	268.03731(100)	ResPect MS/MS
32	Syringaresinol glucoside	580.21559	579.2094	1.1	4.31	417.15576(100)	Sun et al., 2016
33	3,4,7-Trihydroxy-5-methoxy-8-prenylflavan 7-O-beta-D-glucopyranoside isomer 2	534.21011	533.2026	−0.3	4.79	491.19208(100);473.18232(90);503.19246(77)	Abu-Reidah et al., 2013a
34	Hydroxybenzoyl dihydroxybenzoyl hexose	436.10056	435.0934	0.2	5.65	297.06184(21);315.07121(20)	Abu-Reidah et al., 2013a
35	Tri-4-hydroxyphenylacetyl glucoside	582.17373	581.1662	−0.2	6.04	175.03999(100)	Abu-Reidah et al., 2013a

*RT, Retention time*.

a *5-Caffeoylquinic acid and 3-caffeoylquinic acid were the major forms*.

b *3,5-Di-O-caffeoylquinic acid was reported as the major form*.

**Figure 4:**
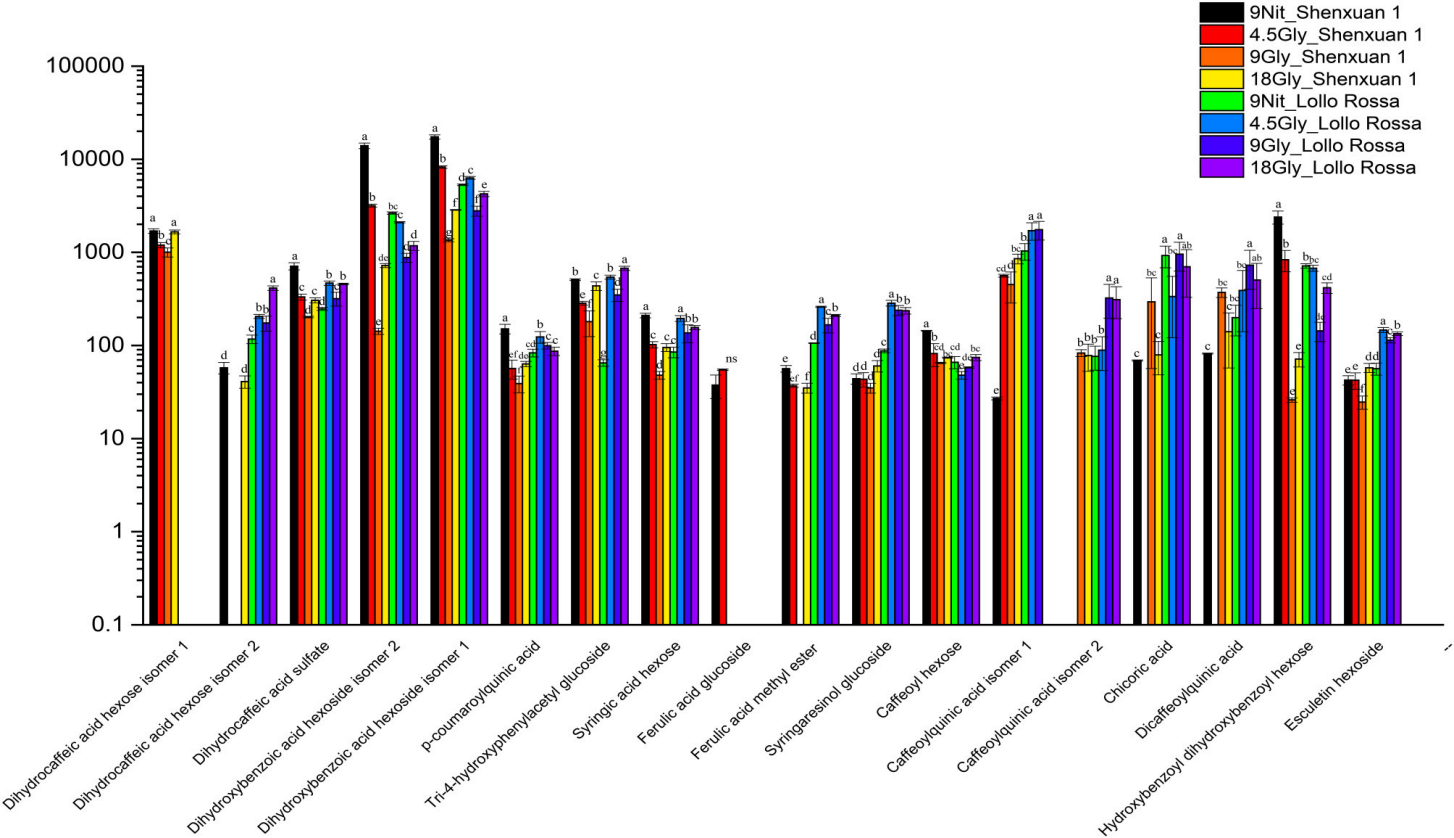
Effects of nitrate and glycine supply on the relative ratios of phenolic acid derivatives in lettuce leaf extracts. Different small letters indicate significant differences (*p* < 0.05) and “ns” indicates non-significant differences (*p* ≥ 0.05; LSD analysis, *n* = 3).

**Figure 5:**
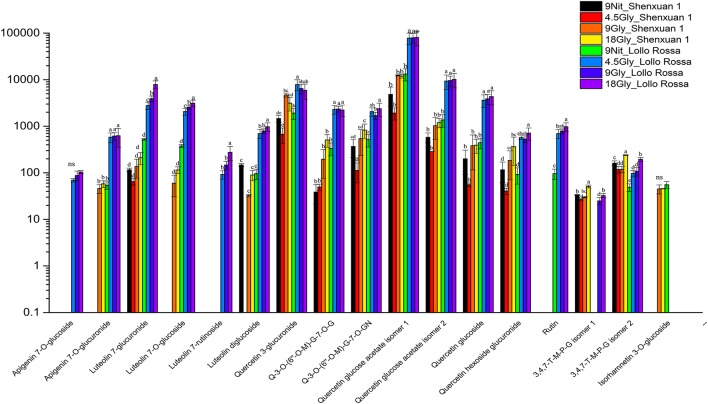
Effects of nitrate and glycine supply on the relative ratios of flavonoid derivatives in lettuce leaf extracts. Different small letters indicate significant differences (*p* < 0.05) and “ns” indicates non-significant differences (*p* ≥ 0.05; LSD analysis, *n* = 3). Q-3-*O*-(6″-*O*-M)-G-7-*O*-G, quercetin 3-*O*-(6″-*O*-malonyl)-glucoside 7-*O*-glucoside; Q-3-*O*-(6″-*O*-M)-G-7-*O*-GN, quercetin 3-*O*-(6″-*O*-malonyl)-glucoside 7-*O*-glucuronide; 3,4,7-T-M-P-G isomer 1, 3,4,7-trihydroxy-5-methoxy-8-prenylflavan 7-*O*-beta-D-glucopyranoside isomer 1; and 3,4,7-T-M-P-G isomer 2, 3,4,7-trihydroxy-5-methoxy-8-prenylflavan 7-*O*-beta-D-glucopyranoside isomer 2.

As shown in Figures 4, 5, the relative contents of apigenin 7-*O*, luteolin 7-*O* and quercetin 3-*O* and caffeoylquinic acids derivatives in Lollo Rossa cultivar are higher than their in Shenxuan 1 lettuce. In the Shenxuan 1 cultivar, glycine supply significantly decreased the contents of dihydroxybenzoic acid derivatives (dihydroxybenzoic acid hexoside isomer 1 and 2), *p*-coumaroylquinic acid, dihydrocaffeic acid sulfate, tri-4-hydroxyphenylacetyl glucoside, ferulic acid methyl ester, caffeoyl hexose, hydroxybenzoyl dihydroxybenzoyl hexose and syringic acid hexose compared to control lettuce cultivated in nitrate; all of these metabolites were present at the highest levels in lettuce cultivated in 4.5 or 18 mM glycine and lowest levels in lettuce cultivated in 9 mM glycine (Figure 4). In addition, apigenin 7-*O*-glucuronide and some luteolin glycoside derivatives (luteolin 7-*O*-glucoside, luteolin 7-glucuronide) and quercetin glycoside derivatives (quercetin 3-glucuronide, quercetin 3-*O*-(6″-O-malonyl)-glucoside 7-*O*-glucuronide, quercetin 3-*O*-(6″-*O*-malonyl)-glucoside 7-*O*-glucoside, quercetin glucose acetate isomer 1 and 2) were not detected in control Shenxuan 1 lettuce, but were induced by 9 and 18 mM glycine (Figure 5).

In the Lollo Rossa cultivar, glycosylated luteolin derivatives, quercetin derivatives, apigenin derivatives, dihydrocaffeic acid derivatives (dihydrocaffeic acid hexose isomer 2, dihydrocaffeic acid sulfate), tri-4-hydroxyphenylacetyl glucoside, syringic acid hexose, ferulic acid methyl ester, syringaresinol glucoside and esculetin hexoside were significantly induced by glycine, whereas the dihydroxybenzoic acid derivatives (dihydroxybenzoic acid hexose isomer 1 and 2) and hydroxybenzoyl dihydroxybenzoyl hexose were significantly reduced by 9 and 18 mM glycine (Figures 4, 5).

### Effect of glycine on antioxidant activity

**Figure 6:**
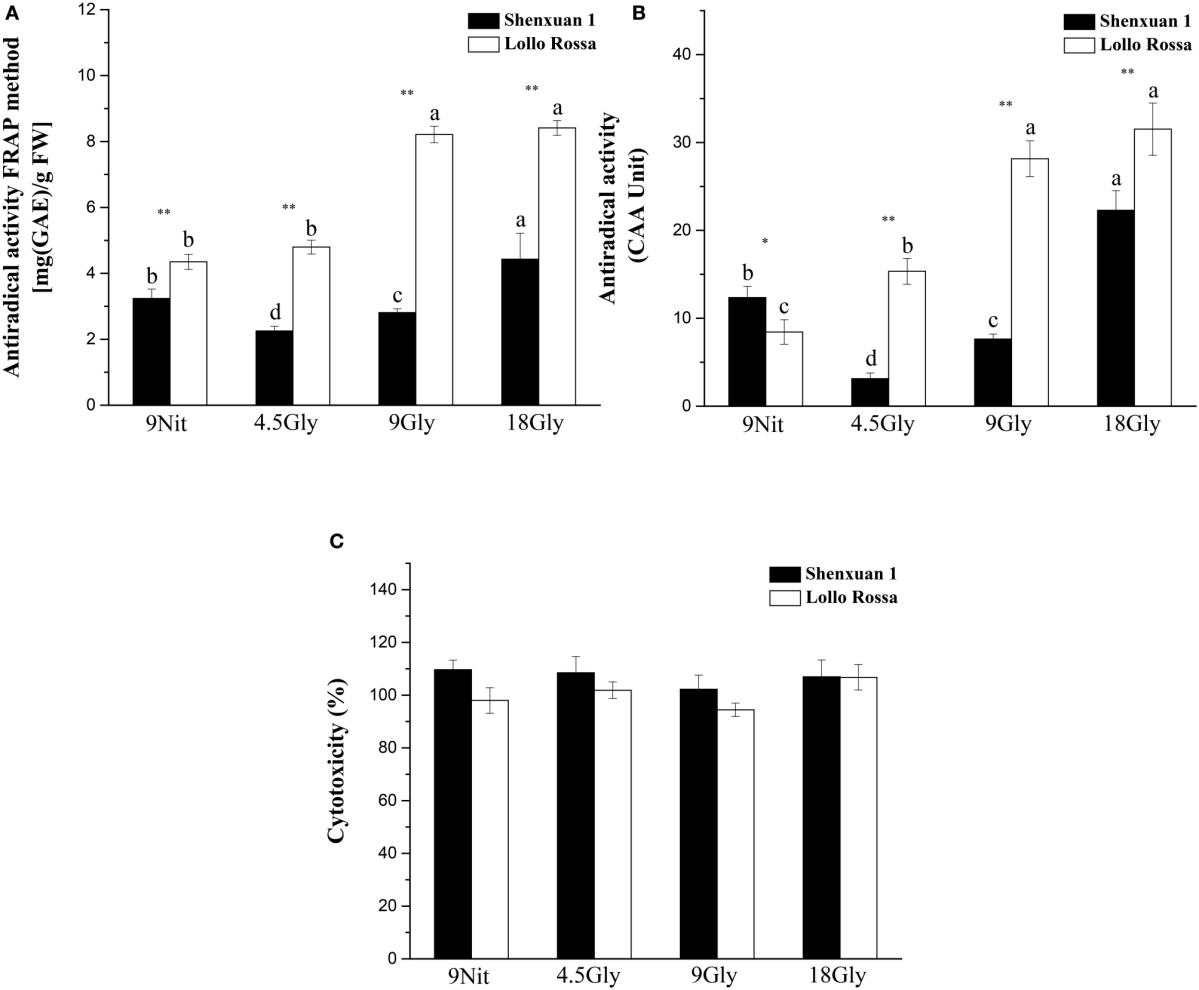
Effect of glycine or nitrate supply on the antioxidant activity of lettuce leaf extracts. **(A)** FRAP assay. **(B)** CAA assay. **(C)** Cytotoxicity assay. Different small letters indicate significant differences, *p* < 0.05; LSD analysis (*n* = 3). The “^*^” (*p* < 0.05) and “^**^” (*p* < 0.01) indicate significant differences between cultivars (*t*-test).

To assess the effect of glycine supply on antioxidant activity, *in vitro* assays were performed to directly evaluate simple ferric reducing ability and cellular antioxidant activity (Figures 6A,B). Both nitrate- and glycine-treated lettuce leaf extracts had low cytotoxicity toward HepG2 cells in the CCK-8 assay (Figure 6C). However, supply of 9 or 18 mM glycine significantly increased the antioxidant activity of the Lollo Rossa cultivar extracts, which peaked in lettuce supplied with 18 mM glycine with a 3.3-fold (CAA) and 1.93-fold (FRAP) increase compared to control plants. In the Shenxuan 1 cultivar, 18 mM glycine significantly increased FRAP by 1.4-fold and CAA by 1.8-fold compared to nitrate-treated control plants (Figures 6A,B).

**Figure 7:**
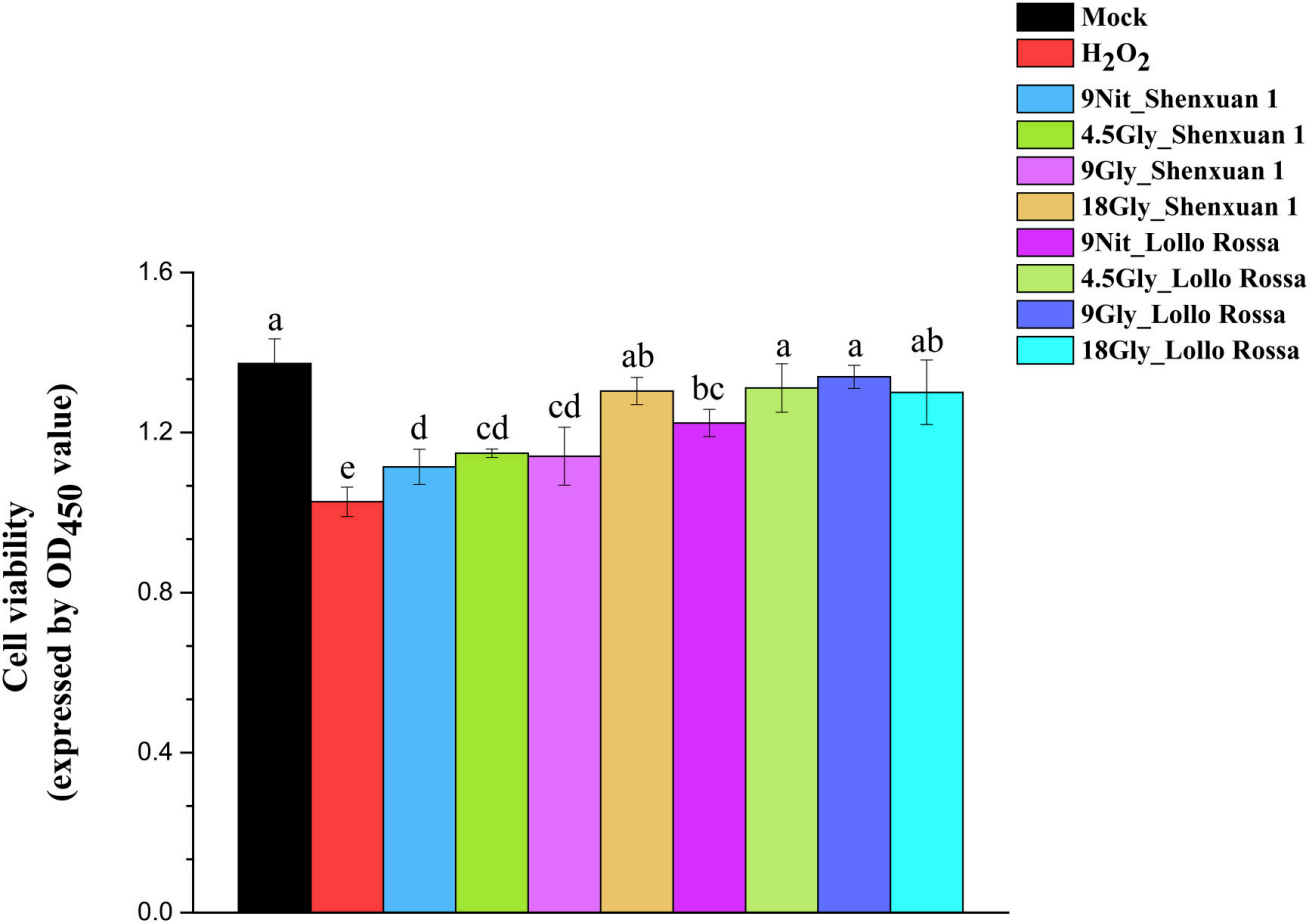
Effect of glycine or nitrate supply on the H_2_O_2_ scavenging ability of lettuce leaf extracts in the CCK-8 assay. Different small letters indicate significant differences, *p* < 0.05; LSD analysis (*n* = 3).

Moreover, the CCK-8 assay was conducted to evaluate the viability of HepG2 cells and verify the effect of extraction on H_2_O_2_ scavenging capability (Figure 7). Cell viability was significantly higher in all treatments compared to H_2_O_2_ treatment, suggesting the extraction process is superoxide-scavenging. In addition, no significant differences were observed between the mock treatments and all Lollo Rossa samples supplied with glycine and Shenxuan 1 samples supplied with 18 mM glycine. Glycine-treated lettuce exhibited higher scavenging capability than nitrate-treated control lettuce. In the Shenxuan 1 cultivar, the cell viability of lettuce exposed to 18 mM glycine was significantly higher than control lettuce, while the extracts of lettuce treated with 4.5 and 9 mM glycine increased cell viability compared to the 9 mM nitrate-treated extracts.

## Discussion

### Glycine nitrogen supply reduces the fresh weight of lettuce plants

The ability of higher plants to use organic nitrogen (amino acids, peptides and proteins) as a nitrogen source has been demonstrated in laboratory studies and field experiments (Näsholm et al., 2009). Compared to nitrogen deficiency, exogenous glycine supply increases production of biomass in *Arabidopsis* plants (Forsum et al., 2008). However, glycine could not support plant growth to the same extent as the same concentration of nitrate in pak choi (Wang, X. et al., 2014). In this study, glycine nitrogen supply significantly decreased the fresh and dry weight of lettuce compared to plants supplied with 9 mM nitrate. In horticulture, 9 mM is suggested as a standard reference concentration in commercial hydroponic lettuce production (Brechner and Both, 2017, Grower's Handbook: Lettuce). It is not surprising that glycine supply decreases plant growth compared to plants provided with the appropriate nitrate concentration in agricultural practice. These results are in accordance with our previous studies of pak choi (Wang X. et al., 2014), which indicated glycine may limit plant root growth (Dominguez-May et al., 2013) and induce differential proteomic responses associated with plant defense or stress and energy and nitrogen metabolism (Wang X. et al., 2014).

### Appropriate concentrations of glycine promote accumulation of antioxidants

Primary antioxidants, such as flavonoids, ascorbic, acid and tocopherols, are abundant in plants, exert various physiological functions (Dixon et al., 2002; Singh and Singh, 2008) and play significant roles in the human diet (Chen and Chen, 2013; Tomas-Barberan et al., 2016). In this study, 18 mM glycine supply significantly (*p* < 0.05) increased total polyphenols and anthocyanidin content compared to 9 mM nitrate or lower concentrations of glycine (4.5 or 9 mM). The influence of nitrogen on the accumulation and bioactivity of antioxidants in plants are controversial. Generally, high levels of inorganic nitrogen (nitrate or ammonium) exert negative, dose-dependent effects on plant flavonoid biosynthesis and anthocyanin accumulation and activity (Patil and Alva, 1999; Awad and de Jager, 2002; Yañez-Mansilla et al., 2014; Becker et al., 2015). In addition to environmental conditions (e.g., temperature, light), the dose-dependent variations observed in plants exposed to different nitrogen sources may also be due to the complexity of plant responses to nutrient availability. According to the growth-differentiation balance hypothesis, plants with sufficient nitrogen supply (e.g., 9 mM nitrate in this study) tend to allocate nitrogen to vegetative growth rather than biosynthesis of phenolics; low supply (e.g., 4.5 mM glycine) limits both growth and secondary metabolite accumulation, In contrast, plants with intermediate resources (e.g., 9 or 18 mM glycine, as N use efficiency is lower for glycine compared with nitrate) accumulate high levels of phenolic acids with an intermediate increase in biomass (Herms and Mattson, 1992; Glynn et al., 2007).

Ascorbic acid is a major antioxidant in lettuce (Nicolle et al., 2004). In this study, ascorbic acid was significantly induced by 9 mM glycine compared to control nitrate and 4.5 or 18 mM glycine. The effects of different concentrations of nitrogen on ascorbic acid synthesis remain controversial. Some studies have indicated increased nitrogen supply increases the vitamin C content in some plants, though most studies reported ascorbic acid decreased or did not significantly change (see review by Mozafar, 1993; Flores et al., 2004). This controversy may be related to inter-plant variations in the optimum nitrogen concentration required for maximal vitamin C accumulation.

Tocopherol is a lipid-soluble natural antioxidant; the α- and γ- forms are the major isomers in lettuce (Nicolle et al., 2004; Cruz et al., 2014). Exposure to 18 mM glycine significantly increased the α-tocopherol content compared to lettuce cultivated in 9 mM nitrate and 4.5 or 9 mM glycine. In both varieties of lettuce, the concentration of γ-tocopherol was significantly higher for plants supplied with 4.5 and 18 mM glycine than control plants. Similarly, previous research reported inorganic nitrogen fertilization increased the concentration of tocopherols in rapeseed, increasing the levels of urea more than the levels of ammonium (Hussain et al., 2014).

### Glycine supply promotes accumulation of apigenin-3-*O*, quercetin-3-*O* and luteolin-7-*O* glycoside derivatives

The phenylpropanoid and flavonoid pathways synthesize a wide range of secondary metabolites including phenolic acid derivatives, lignins and flavonoids, which play important roles in both plant growth and human nutrition (Tzin and Galili, 2010). Glycine supply decreased the contents of several phenolic acids (e.g., hydroxycinnamic and hydroxybenzoic derivatives in the Shenxuan 1 cultivar; hydroxybenzoic derivatives in Lollo Rossa), but led to accumulation of luteolin, apigenin and quercetin glycoside derivatives. In general, phenolic acid derivatives and flavonoid biosynthesis share the same precursor, *p*-coumaroyl CoA. The induction of flavonoid biosynthesis and reductions in the content of some phenolic acids and derivatives observed in the presence of glycine indicate altered precursor availability induced metabolic flux from phenolic acid biosynthesis to flavonoid pathways by altering the expression of chalcone synthesis and auxin polar transport (Besseau et al., 2007; Taulavuori et al., 2016). In addition, glycine significantly promoted the accumulation of sugars, which may positively stimulate the biosynthesis of flavonol glycosides by increasing the supply of carbon rings and glycosides (Liu et al., 2016).

Luteolin and quercetin derivatives have a greater capacity to scavenge ROS than most other flavonoids (Brunetti et al., 2013), thus an increase in the luteolin to apigenin glycosides ratio and kaempferol to quercetin glycosides ratio are a component of plant responses to light quality and intensity; luteolin (or quercetin) glycoside derivatives increased significantly, while apigenin (or kaempferol) glycosides derivatives increased only slightly in response to light (Markham et al., 1998; Tegelberg and Julkunen-Tiitto, 2001; Oh et al., 2009). In this study, apigenin glycosides were not detected (Shenxuan 1) or only present at trace levels (Lollo Rossa) in the control lettuce supplied with nitrate, whereas a high concentration of glycine (18 mM) induced accumulation of apigenin glycosides. In addition, the downstream metabolites luteolin glycoside derivatives and another dihydroxy B-ring-substituted flavonoid (quercetin 3-*O* glycoside derivatives) were also significantly induced by glycine compared to control lettuce. For example, 15-fold (Lollo Rossa) and 2-fold (Shenxuan 1) increases in luteolin 7-glucuronide were observed in lettuce supplied with 18 mM glycine compared to the respective control lettuce supplied with 9 mM nitrate. Moreover, 10- and 3-fold increases in quercetin glucoside were observed in Lollo Rossa and Shenxuan 1 supplied with 18 mM glycine compared to control lettuce. Thus, we hypothesize that apigenin-3-O, quercetin-3-O, and luteolin-7-O glycoside derivatives may represent signals of the response to glycine supply and indicate a metabolic switch from accumulation of small quantities of glycosylated flavonoids to synthesis of both monohydroxy and dihydroxy B-ring-substituted flavonoid derivatives.

### Appropriate concentrations of glycine promote antioxidant bioactivity

Genotype and growing conditions influence antioxidant compositions and bioactivity in lettuce. Red leafed lettuce cultivars have higher average total polyphenol contents and antioxidant capacities than green leafed cultivars (Liu et al., 2007). In this study, extracts from the Lollo Rossa cultivar exhibited significantly stronger ferric-reducing antioxidant power, cellular antioxidant activity and H_2_O_2_ scavenging ability than the Shenxuan 1 cultivar. The Lollo Rossa cultivar is likely to contain significantly higher levels of polyphenols (particularly glycosylated quercetin, apigenin, and luteolin), vitamin C and anthocyanins, which correlate positively with antioxidative activity.

**Figure 8:**
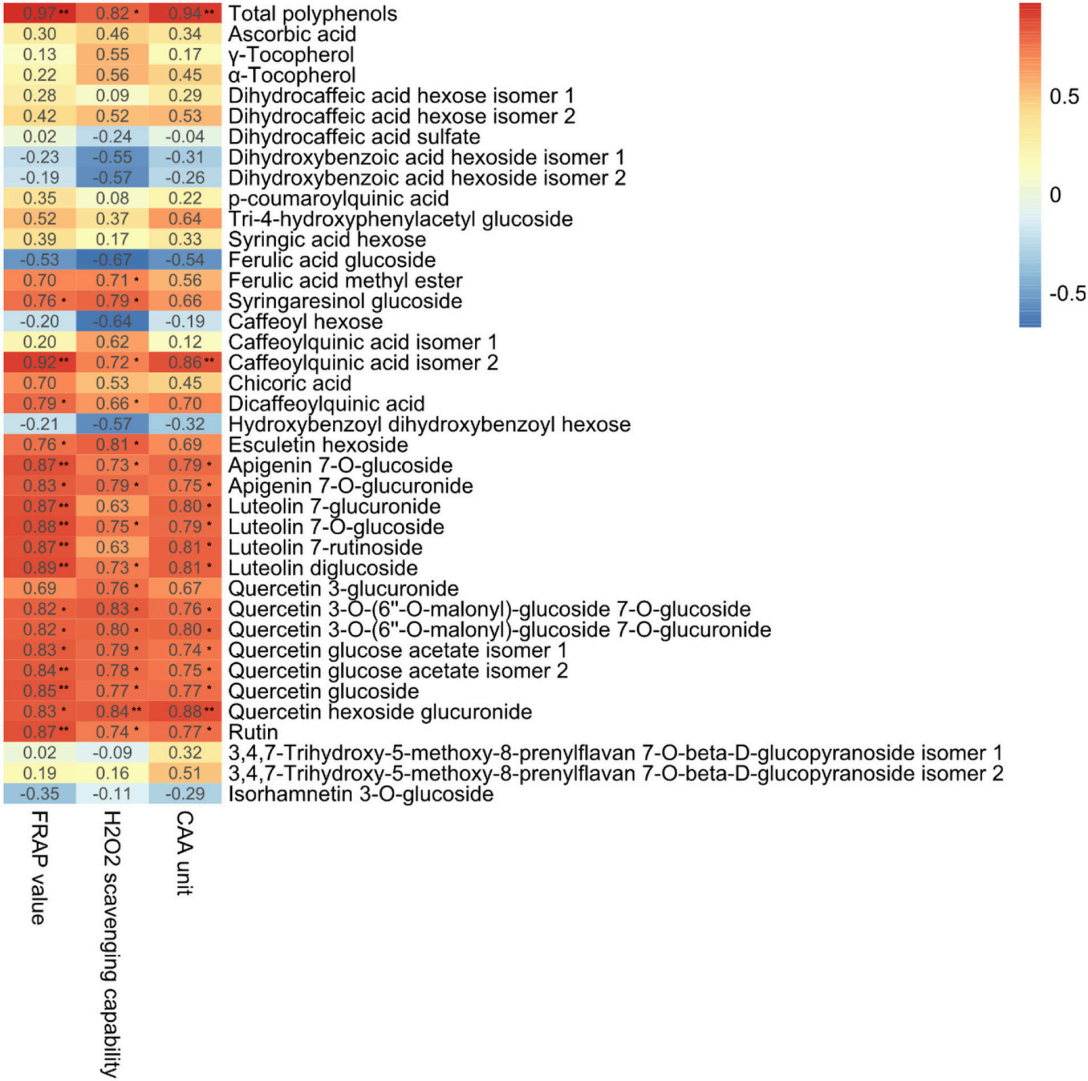
Correlation coefficients (*r*) for the relationships between total antioxidant capacity and total polyphenols and individual polyphenols in the leaves of lettuce hydroponically cultivated in media containing glycine or nitrate. ^**^p < 0.01 and ^*^0.01 < *p* < 0.05; two-tailed test.

We performed Pearson Correlation analysis to investigate the possibility of an inter-relationship between the metabolites detected and antioxidant activity, as indicated by FRAP, CAA, and H_2_O_2_ scavenging capability (Figure 8). Antioxidant bioactivity was significantly (*p* < 0.05) and positively (*r* > 0.75) correlated with total polyphenol content and the levels of apigenin 7-*O*-glucuronide, luteolin 7-glucoside, quercetin 3-*O*-(6″-*O*-malonyl)-glucoside 7-*O*-glucoside, quercetin 3-*O*-(6″-*O*-malonyl)-glucoside 7-*O*-glucuronide, quercetin glucose acetate isomer 2, quercetin glucoside and quercetin hexoside glucuronide. These results are in agreement with a previous study of *Stevia rebaudiana* leaves treated with nitrogen, which found antioxidant bioactivity positively correlated with total phenolic acids and the levels of glycosylated quercetin, apigenin, and luteolin (Tavarini et al., 2015).

Glycine-treated lettuce extracts exhibited higher scavenging capability than nitrate-treated control lettuce extracts. The antioxidant bioactivity of Shenxuan 1 lettuce exposed to 18 mM glycine was significantly higher than that of control lettuce, while the extracts of lettuce treated with 9 and 18 mM glycine had higher antioxidative activities than the 9 mM nitrate-treated extracts. These results can mainly be attributed to the significantly higher total levels of polyphenols, particularly luteolin, quercetin and apigenin glycosides, in the lettuce treated with 18 mM glycine. A luteolin or quercetin-rich diet is related to reduced risks of specific types of cancer (Ekström et al., 2010; Lam et al., 2012; Lin et al., 2014) and cardiovascular disease (Duthie et al., 2000; Lee et al., 2011), and plays a protective effect in diabetes (Babu et al., 2013). Thus, exogenous glycine supply may promote the accumulation of health-promoting compounds and increase the antioxidative activity of lettuce, which could potentially be beneficial for human nutrition.

## Conclusion

The appropriate concentration of glycine (18 mM for Shenxuan 1; 9 mM for Lollo Rossa) significantly enhanced the levels of antioxidants, including total polyphenols and α-tocopherol, and antioxidant activity (as indicated by FRAP, CAA, and H_2_O_2_ scavenging capability) compared to lettuce supplied with nitrate. Most glycosylated flavonoids detected, including apigenin, quercetin and luteolin, were also induced by 9 and 18 mM glycine, whereas glycine decreased the levels of some phenolic acids. This study indicates exogenous glycine supply could be used strategically to promote the accumulation of health-promoting compounds and increase the antioxidative activity of hydroponically grown lettuce; this strategy may have potential relevance to human nutrition.

## Author contributions

XY, XC, and LZ performed all the experimental measurements, analyzed the data, and drafted the manuscript. DG, LF, and SW helped with the figures and samples. CZ and DH designed experiment and supervised all the results, and contributed to writing the manuscript.

### Conflict of interest statement

The authors declare that the research was conducted in the absence of any commercial or financial relationships that could be construed as a potential conflict of interest.
