# Supplementary figures and images for: Exogenous Glycine Nitrogen Enhances Accumulation of Glycosylated Flavonoids and Antioxidant Activity in Lettuce (Lactuca sativa L.)
Source: Front Plant Sci. 2017 Dec 15;8:2098. doi: 10.3389/fpls.2017.02098 (PMC5737139; doi:10.3389/fpls.2017.02098)

**Supplemental Figure 1. PCA scores for UPLC-MS samples**

**
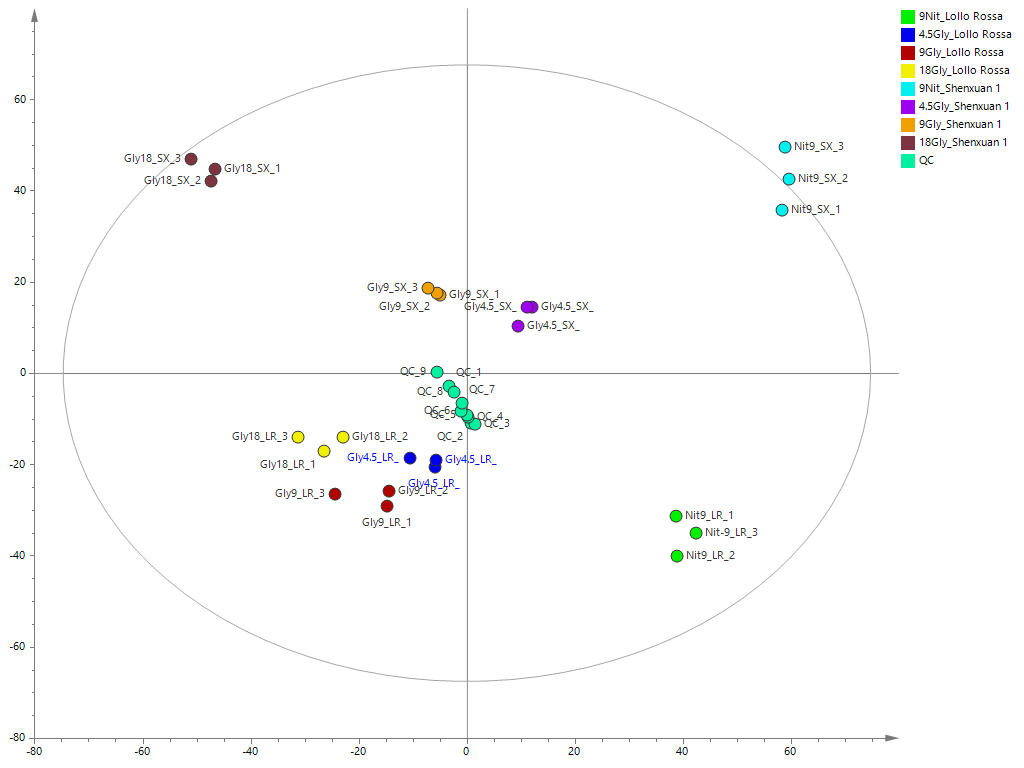
**

Supplement: Supplementary file 1 [file DataSheet1.docx]
